# Fungal vincristine from *Eutypella* spp - CrP14 isolated from *Catharanthus roseus* induces apoptosis in human squamous carcinoma cell line -A431

**DOI:** 10.1186/s12906-016-1299-2

**Published:** 2016-08-22

**Authors:** Gini C. Kuriakose, Padmini P. C. Palem, Chelliah Jayabaskaran

**Affiliations:** Department of Biochemistry, Indian Institute of Science, Bangalore, 560 012 India

**Keywords:** *Eutypella* spp - CrP14, Fungal vincristine, Squamous carcinoma cells, A431, Apoptosis

## Abstract

**Background:**

*Catharanthus roseus,* a medicinal plant, is known to produce secondary metabolites, vincristine and vinblastine, which are terpenoid indole alkaloids. Previously we have reported that *Eutypella* spp – CrP14 isolated from stem cutting of this plant had shown significant antiproliferative activity when tested in vitro against HeLa cell line. The present study was conducted to identify the anticancer compound responsible for the anti-proliferative activity of the fungal extract and to evaluate its in vitro anticancer and apoptotic effects.

**Methods:**

The anti-proliferative activity of the fungal anticancer compound, vincristine was analyzed by MTT assay against different cancer cell lines. We examined its efficacy of apoptotic induction on A431 cells. The parameters examined included cell cycle distribution, loss of mitochondrial membrane potential (MMP), DNA fragmentation and reactive oxygen species (ROS) generation.

**Results:**

The presence of vincristine in fungal culture filtrate was confirmed through chromatographic and spectroscopic analyses, and the amount was estimated to be 53 ± 5.0 μg/l. The partially purified fungal vincristine had strong cytotoxic activity towards human squamous carcinoma cells – A431 in the MTT assay. Furthermore, we showed that the fungal vincristine was capable of inducing apoptosis in A431 cells through generation of reactive oxygen species and activation of the intrinsic pathway leading to loss of MMP.

**Conclusions:**

We have demonstrated for the first time that the vincristine from *Eutypella* spp – CrP14 is an efficient inducer of apoptosis in A431 cells, meriting its further evaluation in vivo.

## Background

Squamous cell carcinoma (SSC) is a common skin cancer in humans and SSC therapy is currently limited to surgery followed by radiation treatment [1]. Chemotherapy is the most sought after treatment in the initial stages with drugs that cause apoptosis of proliferating cells. Several side effects such as drug resistance and non-targeted cell death as well as high cost are some of the constraints in deploying these drugs. Hence, the need for exploration of unconventional natural means had been rightly emphasized [2]. Bioactive natural products from plants and microorganisms with minimal side-effects are encouraged as an alternative.

A large number of secondary metabolites are produced by microorganisms [3]. The list of endophytic fungi producing biologically active compounds of pharmaceutical relevance, an unexplored field [4], is expanding every year. Some of these bioactive metabolites, may be specific or non-specific to the host plant, and are known to have anticancer as well as other therapeutic actions [5]. The ‘million dollar’ drug Taxol, isolated from *Taxomyces andreanae* [6] is a typical example. Some endophytic fungi are now found to be sources of vincristine, camptothecin, taxol, podophyllotoxin, and others [7], originally obtained from the host plants.

*Catharanthus roseus* is a valuable medicinal plant belonging to Apocynacea family and extensively used the world-over in traditional herbal medicine. Vincristine and vinblastine, the two major anticancer vinca alkaloids in chemotherapy regimens, are produced in this plant. The production of these compounds in *C. roseus* is limited. In order to obtain 1 g of these compounds, 500 kg of dried leaves should be processed [8]. It is estimated that 3 kg of vinca alkaloids are required annually world - wide for treating various types of cancers. Considering the limitations associated with extraction and production protocols available till date, and also the high prices from $ 1 million to $ 3 million per kg, a widespread research interest should be initiated in finding alternative novel sources for vinca alkaloid production [9]. Earlier, we have isolated 22 endophytic fungi from various tissues of *C. roseus* and reported the production of vincristine and vinblastine by *Talaromyces radicus* [10]. Additionally, we found that the culture extract of one of the isolates, *Eutypella* spp - CrP14 exhibited strong anti-proliferative activity in HeLa cells. In the present investigation, we identified vincristine as the anticancer compound produced by *Eutypella* species by using chromatographic and spectroscopic techniques and by comparison with the authentic reference standard. We also report that the FVCR induces apoptosis in A431 cells in vitro.

## Methods

### Chemicals and reagents

Vincristine sulphate and Dragendorff’s reagent were procured from Sigma Aldrich (USA). Dimethyl sulfoxide (DMSO), Dulbecco’s modified eagle medium (DMEM), Sodium dodecyl sulfate (SDS), Tris–HCl, Ethylenediaminetetraacetic acid (EDTA), DAPI, Ethidium bromide, Fetal bovine serum, Propidium iodide, RNase A, Proteinase K, 2,4-DNP were obtained from Sigma Chemicals Company (San Diego, USA). DCFH-DA and Annexin-V stains were obtained from Life Technologies (USA). JC-1 (5, 5′, 6, 6′-tetrachloro-1, 1′, 3, 3′-tetraethylbenzimidazolyl carbocyanine iodide) dye was purchased from Molecular probes (Eugene, OR, USA). All other reagents and chemicals were of analytical grade.

#### *Eutypella* fungal culture

The fungus used in this study was isolated from stem cuttings of *C. roseus* plant collected from the nursery of the Indian Institute of Science, Bangalore, India. This fungus was identified as *Eutypella* species. (Order: Xylariales, Family: Diatrypaseae) on the basis of sequence data which included the 5.8 s rDNA gene and ITS genes. The partial ITS sequence was deposited in NCBI GenBank with accession no. KC920840. The fungus was maintained on potato dextrose agar at 25 °C ± 2 °C with regular sub-culturing.

#### Cultivation of *Eutypella* spp – CrP14 and extraction of vincristine

The endophytic fungus was grown on PDA plates for about 10 days. Three 5 mm agar plugs containing mycelium of *Eutypella* spp were transferred to 400 ml of potato dextrose broth, in a 1 L conical flask and incubated at 25 °C ± 2 °C under stationary condition for 21 days. After completion of incubation, the culture was passed through two layers of muslin cloth, in order to get a clear filtrate devoid of mycelia. For alkaloid extraction, a modified protocol was used, based on the methods described elsewhere [10–12]. The pH of the filtrate was adjusted to 2.0 with 1 N H_2_SO_4_ and extracted with equal volume of ethyl acetate. The aqueous phase obtained was retained and the pH was adjusted to 10.0 with 1 N NaOH, and again extracted with equal volume of ethyl acetate. The organic phase was evaporated to dryness at 35 °C using rotary vacuum evaporator and the extract was subsequently used in the experiments.

### Analysis of fungal vincristine (FVCR) from *Eutypella* spp-CrP14

Thin layer chromatography (TLC) analysis was carried out on Merck 0.25 mm silica gel plates. The fungal filtrate culture extract as well as standard vincristine were chromatographed concurrently on a TLC plate and developed in solvent chloroform: methanol (7:3). Upon completion, the fungal vincristine (FVCR) and also the reference standard on TLC plate were visualized by spraying with alkaloid-specific Dragendorff’s reagent.

Mass spectroscopy was done on the fungal filtrate culture extract using the electrospray ionization technique. Fungal extract and also a standard vincristine (SVCR) solution were infused separately into the mass spectrometer through a reverse-phase C18 column (BDS Hypersil), 250 mm × 4.6 mm. The mobile phase used was acetonitrile: 1 % acetic acid (20: 80) at a flow rate of 0.5 ml / min and the data was acquired over a m/z range of 200–100 in positive ion mode.

Quantification of FVCR was performed by HPLC analysis. Fungal filtrate culture extract was subjected to HPLC separation using Agilent C18 column (4.6 × 150 mm, 3 μm pore size and particle size of 120 A) in a Thermofischer PDA detector with a wave length of 252 nm. A gradient elution of 10 % - 100 % was performed using 0.1 % formic acid in 5 mM ammonium acetate (solution A) and methanol (solution B) at a flow rate of 0.8 ml/min. A sample of 20 μl was injected and analyzed. The identification of FVCR was accomplished by comparison of retention times with authentic standard vincristine.

### Apoptotic activity of FVCR

#### Preparation of FVCR

The fungal extract prepared from *Eutypella* spp-CrP14 was first separated on TLC. The spot corresponding to vincristine in crude was scraped and eluted twice with methanol. The resulting partially purified material was used as ‘fungal vincristine’ in apoptotic studies.

#### Cell lines and maintenance

The cell lines A431, HeLa, A549 and HEK 293 were procured from National Centre for Cell Sciences (NCCS) Pune, India. Cells were cultured in Dulbecco’s Modified Eagle’s Medium (DMEM) supplemented with 10 % fetal bovine serum (FBS) and penicillin + streptomycin (100 μg/ml each) mixture in a 5 % CO_2_ incubator at 37 °C. The cell lines were maintained with regular passaging.

#### Antiproliferative activity of FVCR on different cancer cell lines

Cells at a density of 1 × 10^4^ cells per well were seeded in 100 μl of Dulbecco’s modified Eagle’s medium (DMEM) with 10 % fetal bovine serum (FBS) in 96-well plates and grown for 24 h at 37 °C in a 5 % CO_2_ incubator. The cells were then treated with FVCR at various concentrations, ranging from 5 to 100 μg/ml. The cells were then treated with FVCR at various concentrations, ranging from 5 to 100 μg/ml. After 24 h, MTT solution (10 μl of a 5 mg/ml stock) in PBS was added to each well and incubated for 2 h; the supernatant was then removed, and DMSO (100 μl) was added to each well to dissolve the formazan crystals. The absorbance was measured at 570 nm using a microplate reader. The most sensitive cell line was further tested with authentic vincristine, which served as positive control. Standard vincristine is known to inhibit cell proliferation in this test system in our laboratory [10].

#### Cytotoxicity of FVCR – Live dead cell assay using PI staining

The assay was performed using propidium iodide (PI), a membrane impermeable dye that is generally excluded from viable cells. It binds to double stranded DNA by intercalating between base pairs. Briefly, A431 cells (1 × 10^6^) were cultured with different concentrations of FVCR (5, 10 and 20 μg/ml) for 24 h and re-suspended in 100 μl phosphate buffer saline (PBS). Propidium iodide (PI) stain (5 μl) was added to each sample just prior to FACScan analysis and data were acquired for unstained and PI stained cells. Dot-plot of forward scatter versus PI was recorded to check the percentage of cell death.

#### Cell cycle analysis to determine cell cycle distribution

A431 cells (2.5 × 10^5^) were seeded in 24-well plates containing a 500 μl culture medium and incubated for 24 h in a 5 % CO_2_ incubator at 37 °C. Cells were then treated with different concentrations (5, 10 and 20 μg/ml) of FVCR and incubated for 24 h. Cells were trypsinized, re-suspended in phosphate buffer saline (PBS), centrifuged for10 min and fixed in ice-cold ethanol (70 % v/v) for 30 min. The cells were then stained with PI and analyzed by flow cytometer as described previously [13]. The data were analyzed using CellQuest Pro software.

#### Detection of apoptosis and cell death using annexin-V-FITC and PI dual staining

The apoptotic cell death induced by FVCR was measured by flow cytometry using the annexin V-FITC/PI staining method [14]. Cells (2.5 × 10^5^) were seeded in 24-well plates and treated with different concentrations (5, 10 and 20 μg/ml) of FVCR for 24 h in the DMEM-FBS medium. The cells were then stained with recommended concentrations of annexin V-FITC with and without PI and quantified through flow cytometry using the FACScan Calibur (Becton Dickinson, USA). Cells were labeled in the four quadrants as follows: Q3-Annexin V negative and PI negative were considered to be viable cells; Q4- Annexin V positive and PI negative as early apoptotic cells; Q2- Annexin V positive and PI positive as late apoptotic cells or necrotic cells; and Q1-Annexin V negative and PI positive as necrotic cells/ dead cells. The data obtained with an excitation (λ_ex_)/emission (λ_em_) wavelengths of 488/520 nm for annexin V-FITC and 540/630 nm for PI were analyzed using CellQuest Pro software.

#### Determination of DNA fragmentation

The DNA fragmentation analysis was carried out by agarose gel electrophoresis. FVCR-treated and untreated A431 cells were harvested and re-suspended in phosphate-citrate buffer (40 μl containing 192 parts of 0.2 M Na_2_HPO_4_ and 8 parts of 0.1 M of citric acid, pH 7.8) and left for 30 min at room temperature. The cells were then centrifuged for 5 min followed by addition of Nonidet P-40 (3 μl of 0.25 %), RNase A (3 μg) and incubated at 37 °C. After 30 min, proteinase K (3 μg) was added and fragmented DNA was precipitated with the addition of ethanol overnight at −20 °C followed by centrifugation. The DNA pellet obtained was dissolved in TE buffer, and analyzed by 0.8 % agarose gel electrophoresis. The fragmented DNA ladder formation in A431 cells was visualized on a UV transilluminator after staining with 5 μg ethidium bromide.

#### Determination of mitochondrial membrane potential (ΔΨm)

The mitochondrial membrane potential was assessed by using JC-1 dye, a lipophilic cationic fluorescent dye with dual (red and green) emission wavelengths. It exists as a monomer (green) in cytosol and accumulates as red aggregates on selective entry into mitochondria in normal cells [15]. In apoptotic and necrotic cells, JC-1 exists in monomeric form and stains green. A431cells (2.5 × 10^5^) were cultured in the presence or absence of different concentrations (5, 10 and 20 μg/ml) of FVCR for 24 h. The cells were then stained with 2.5 μg/ml of JC-1 dye and incubated for 15 min at 37 °C in a CO_2_ incubator. At the end of incubation, the cells were washed with ice-cold PBS containing FBS (2 % v/v), and both red and green fluoresence were analysed with a flow cytometry using FACS Calibur (Becton Dickinson, USA). JC-1 monomers emit at 530 nm (channel-green fluorescence) and JC-1-aggregates emit at 590 nm (channel-red fluorescence). Valinomycin-treated (10 μM) cells served as the positive control, and untreated cells were taken as the negative control. Data were analyzed using CellQuest Pro software.

#### Measurement of ROS production

The involvement of ROS in FVCR induced apoptosis pathway was studied using the dye 2, 7-dichlorodihydrofluorescein diacetate (DCFH-DA) [16]. Briefly, A431 cells (2.5 × 10^5^) were seeded in 24-well plates, incubated for 24 h and treated with different concentrations (5, 10 and 20 μg/ml) of FVCR for 24 h in DMEM-FBS medium. In order to detect the production of ROS, the cells were harvested, washed with PBS, re-suspended in PBS containing DCFH-DA (10 mM) and incubated at 37 °C for 30 min. Hydrogen peroxide (800 μM) was used as a positive control. Fluorescence generated due to the hydrolysis of DCFH-DA to dichlorodihydrofluorescein (DCFH) by non-specific cellular esterases and the subsequent oxidation of DCFH by peroxides was measured by flow cytometry for green fluorescence at 530 nm.

#### Statistical analysis

All data were expressed as a mean ± standard deviation of three independent experiments.

## Results

### Identification of vincristine in the culture extract of *Eutypella* spp-CrP14

**Fig. 1:**
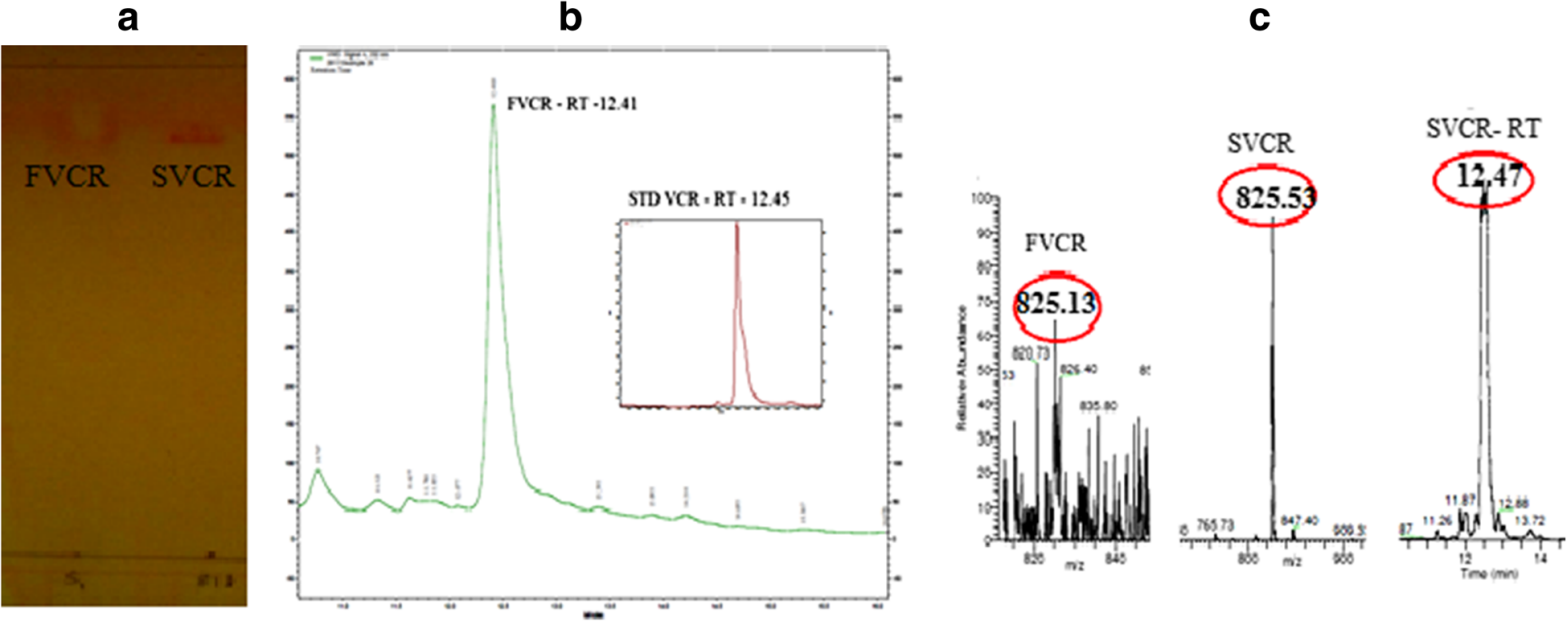
Identification of vincristine isolated from *Eutypella* spp-CrP14 culture medium. **a** TLC analysis of the fungal culture filtrate extract on a silica gel aluminium sheet. The chromatogram was developed using chloroform:methanol (7:3 v/v) and sprayed with Dragendorff’s reagent. Arrows indicate fungal vincristine (FVCR) and standard vincristine (STD VCR) spots. **b** HPLC chromatogram of TLC purified FVCR. **c** LC-ESI-MS analysis of the fungal culture filtrate extract and standard VCR

Previously we have reported that *Eutypella* spp-CrP14 isolated from *C. roseus* possessed excellent anti-proliferative activity against HeLa cells (IC_50_ 13.5 μg/ml) [10]. Conditions of fungal culture were optimized for growth and production of alkaloids. The highest mycelium biomass was observed on day 18 (data not shown). Both the mycelium and the culture filtrate were assessed for the presence of vincristine by chromatographic and spectroscopic analyses. The crude culture filtrate extract separated on silica gel TLC showed the presence of a compound with a characteristic light brown spot with Dragendorff-reagent at the same Rf value as authentic vincristine (Fig. 1a). The mycelia extract did not show any trace of vincristine (data not shown). Separation of the fungal culture filtrate extract on a HPLC reverse phase C18 column eluted a peak with a retention time of 12.41 min that was similar to authentic vincristine (Fig. 1b). Further, evidence on the identity of vincristine was obtained by electrospray ionization mass spectroscopy. Both authentic vincristine and the fungal product showed the characteristic peak at [M + H^+^] 825.5 (Fig. 1c). These results show that the fungal compound is vincristine and the fungus produced 53 ± 5.0 μg/l of vincristine in liquid culture under these conditions.

### Anti-proliferative activity of FVCR

FVCR inhibited cell proliferation in three different types of cancer cell lines - A431, HeLa and A549 cells. We first examined the effect of FVCR on cell proliferation by MTT assay, a convenient method for assessing drug sensitivity. Growth of all the three investigated cell lines, but not the normal cell line HEK 293, was significantly inhibited by FVCR in a dose-dependent manner. The IC_50_values of FVCR were as follows: 4.8 ± 0.33 μg/ml for A431, 10.0 ± 0.25 μg/ml for HeLa and 14.0 ± 0.17 μg/ml for A459 respectively. A431 was more sensitive among the three and was therefore further treated with different concentrations of standard vincristine. An IC_50_ value of 8.0 ± 0.65 nM was obtained for for A431 and therefore used in further experiments.

### Cytotoxicity of FVCR in A431 cells

**Fig. 2:**
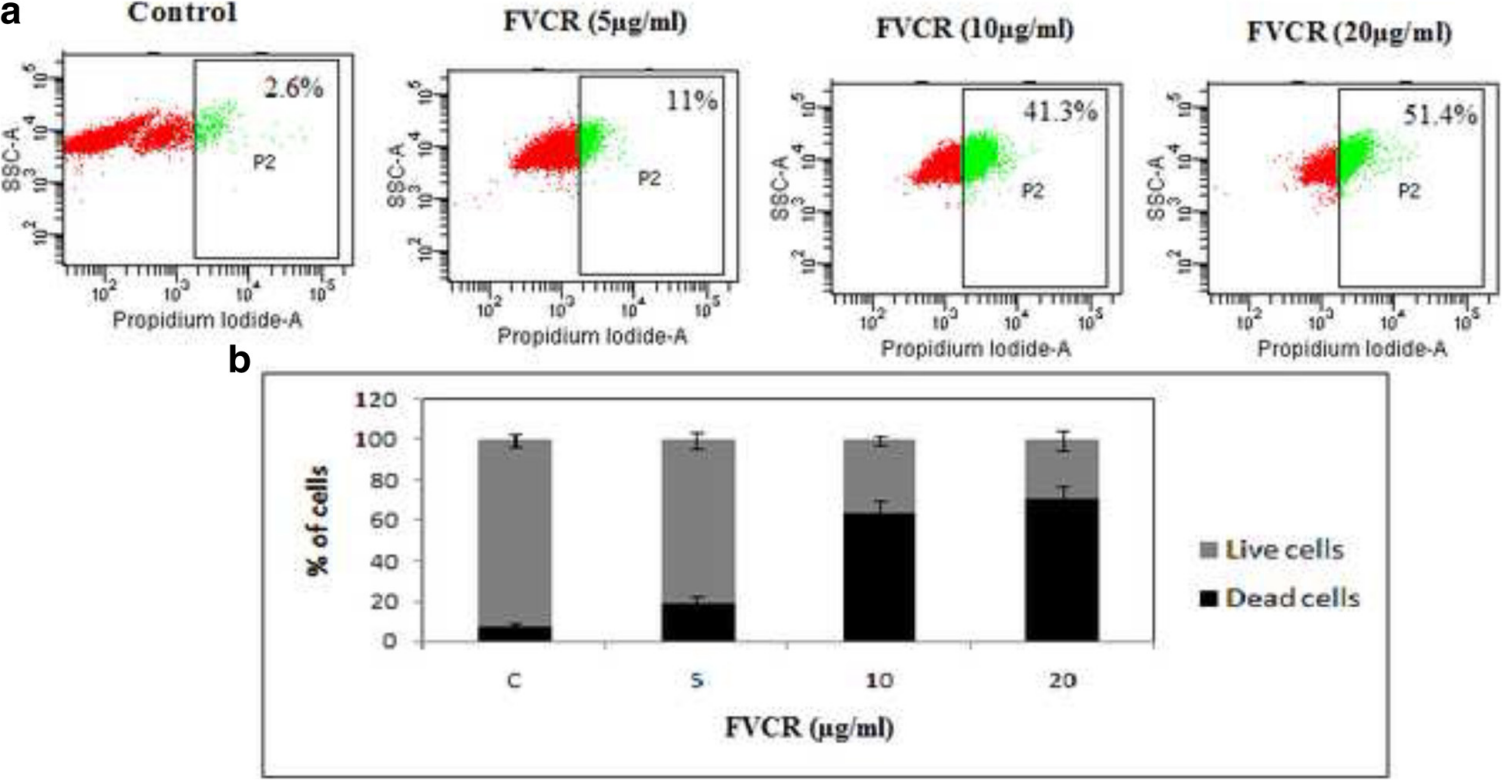
Cytotoxic activity of FVCR in A431 cells. **a** Evaluation of cytotoxic effect A431 cells treated with different concentrations of FVCR stained with PI for 24 h and subjected to flow cytometric analysis to evaluate live and dead cell population. **b** Bar diagram shows the % of live and dead cells present in the FVCR-treated and untreated A431 cells

The live-dead cell assay was performed to study the cytotoxic effect of FVCR on proliferation of A431 cells. The cells treated with different concentrations of FVCR, stained with PI and subjected to FACS analysis, showed concentration-dependent effect on viability after 24 h treatment with respect to dead cell population when the Dot-plot of forward scattered versus PI was recorded. The percent dead cells were found to be 2.6 ± 0.25 in control and 11.0 ± 0.13, 41.3 ± 0.32 and 51.4 ± 0.42 respectively, when treated with 5, 10 and 20 μg/ml of FVCR (Fig. 2a and b). These observations confirm cytotoxicity of FVCR in A431 cells.

### Induction of apoptosis in A431 cells by FVCR

**Fig. 3:**
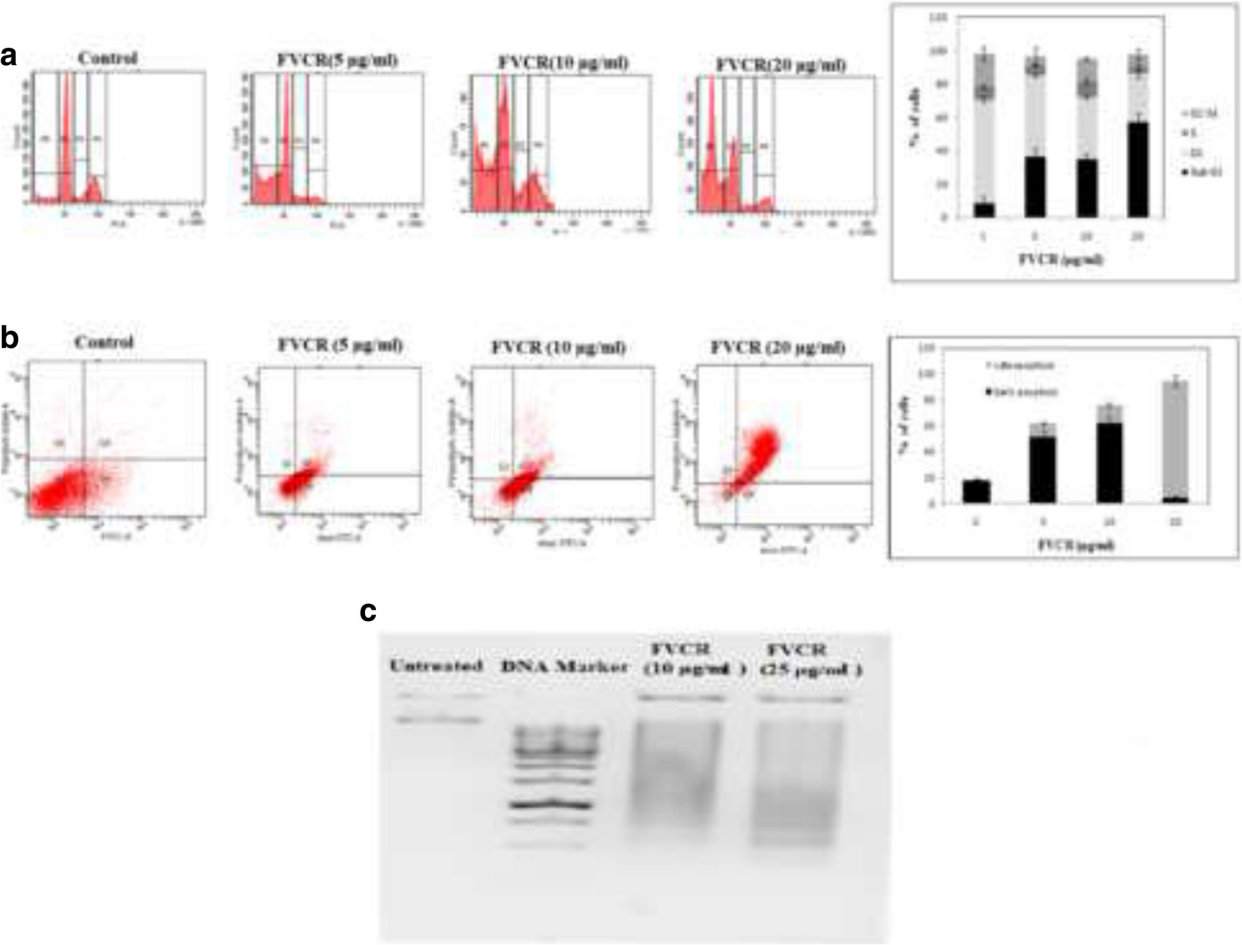
Apoptosis and DNA damage induced by FVCR in A431 cells. **a** Cells were treated with indicated concentrations of FVCR for 24 h and subsequently analysed by flow cytometry using PI staining to determine the populations with hypodiploid DNA (sub G1 phase). The sub-G0/G1, G1, S and G2/M phases are represented on the histogram as P5, P2, P4 and P3, respectively. Quantitative data evaluated by the sub-G1 population of individual histograms. The percentage of apoptotic cells (sub G0/G1 peak) was quantified and plotted against the concentrations of FVCR. **b** Increase in apoptosis was determined by annexin V/PI double staining in A431 cells, at 24 h after FVCR treatment. Bar diagram shows the % of early and late apoptotic cells in FVCR-treated cells. **c** FVCR-induced degradation of chromosomal DNA into small internucleosomal fragments was evidenced by the formation of 180–200 bp DNA ladders on agarose gel

Cell cycle changes of growth arrest and apoptosis in A431 cells treated with FVCR were determined by flow cytometry. After 24 h of treatment with 0, 5 10 and 20 μg/ml of FVCR, a dose-dependent increase in sub G0/G1 cells was observed as compared with that of untreated cells with a high percentage found at a dose of 20 μg/ml (Fig. 3a). Apoptosis is a stage dependent process and annexin V-FITC is primarily employed to detect early/mid stages in cells treated with drugs. The results indicated that FVCR at a low concentration of 5 μg/ml was able to induce 51 % apoptosis and 62 % at 10 μg/ml (Q4). But as the concentration increased to 20 μg/ml, about 90.2 % of cells appeared in late apoptotic stage (Q2), with only 4.3 % of cells showing early apoptosis (Q4). In terms of late apoptosis/necrosis, as the concentrations increased, more cells started accumulating in quadrant Q2 in a dose dependent manner with a drastic leap from 10 to 20 μg/ml (Fig. 3b). We further investigated whether FVCR (10 and 25 μg/ml) could induce DNA fragmentation in A431 cells by the analysis of DNA content by flow cytometry. The agarose gel electrophoresis of DNA of FVCR-treated A431 cells revealed inter-nucleosomal DNA fragmentation characteristic of apoptotic cells (DNA ladder) (Fig. 3c) showing apoptosis in these cells.

**Fig. 4:**
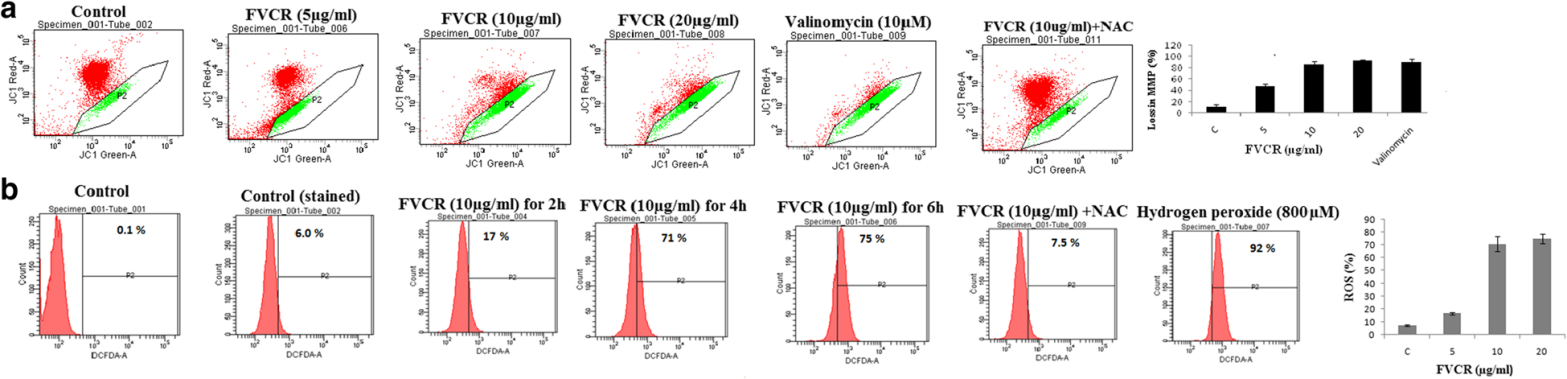
Effect of FVCR on loss of mitochondrial membrane potential and ROS. Cells were treated with different concentrations of FVCR for 24 h and subsequently analyzed for. **a**: Changes in mitochondrial membrane potential in FVCR treated A431 cells as determined by JC-1 staining and detected by flow cytometry analysis. Bar diagram shows the percentage of cells having low mitochondrial membrane potential in the FVCR-treated cells. **b**: ROS production in FVCR-treated cells using DCFH-DA fluorescent probe followed by flow cytometry. Bar diagram shows the percentage of ROS production levels of FVCR-treated cells in a concentration-dependent manner

The loss of mitochondrial membrane potential (ΔΨm), a hallmark for apoptosis, is an early event coinciding with caspase activation. ΔΨm was measured from the shift in the ratio of red to green fluorescence emitting cells following treatment with FVCR. Most of the treated-A431 cells showed green fluorescence in a dose-dependent manner, indicating depolarization and collapse of trans-membrane potential in mitochondria of these cells. We tried to understand the effect of FVCR on depletion of MMP in A431 cell line. Results indicated that FVCR is capable of bringing apoptotic death through intrinsic pathway due to loss of MMP in A431 cells. As the concentrations of the FVCR increased, the accumulation of cells in P2 quarter increased which could be noticed by the increase in green fluorescence intensity. About 47.5, 86.4 and 93.3 % of cells underwent loss in MMP when treated with 5, 10 and 25 μg/ml of FVCR respectively, while Valinomycin (10 μM) recorded 91 % (Fig. 4a). Generation of ROS in A431 cells after treatment with different concentrations of FVCR was measured using DCFH-DA through flow cytometry. Upon treating A431 cells for 24 h with FVCR, a concentration-dependent increase of ROS production was observed (Fig. 4b). The median fluorescence intensity value of control cells was 83 whereas the median values changed to 274, 558 and 705, respectively, when cells were treated with indicated concentration of FVCR at 2 h, 4 h and 6 h time periods. On the other hand, in positive control, where the cells were treated with H_2_O_2_, the median fluorescence intensity value was recorded to be 765. Therefore, comparatively, the FVCR induces higher ROS production in A431 cells.

## Discussion

To the best of our knowledge, this is the first report of vincristine producing endophytic fungus, *Eutypella* spp-CrP14 isolated from the *C. roseus* plant. *Eutypella,* a less studied genus, was found to be pathogenic causing cankers in grape vines [17–19], maple [20] and also as saprobe of woody angiosperms [21]. Recently, it was also isolated from sea sediment [22] as well as an endophyte from a few pharmaceutically important plants [23, 24].

Previously it was shown that VCR promotes mitotic cell cycle arrest at the G2/M phase and also causes apoptosis in many types of cancer cells [25]. In acute lymph oblastic leukemia cells, VCR induced apoptosis through activation of caspase-3 and 9 [26]. However, despite the studies demonstrating apoptotic effect of the combined treatment of VCR along with cisplatin and bleomycin on squamous carcinoma cells [27], the mechanism involved in squamous carcinoma cells is not clear.

The present study demonstrates the potential of *Eutypella* spp-CrP14 to produce vincristine that can induce apoptosis in A431 cells in vitro. Apoptosis plays a crucial role in the normal development of a wide variety of tissues [28]. Most cancer cells do not undergo apoptosis due to impairment of transmission of apoptotic signals [29]. Methods and reagents are now available to identify induction, early, intermediate and late-stages of apoptosis and also to distinguish them from necrotic processes. We adapted some of these techniques to assess the apoptotic potential of the FVCR. The observed accumulation of cells in sub Go/G1 phases in cell cycle distribution indicated induction of early apoptosis in FVCR-treated A431 cells.

Casting of phosphatidylserine from inner to outer phospholipid bilayer of the cell membrane is an early apoptotic event [30]. This externalization of phosphatidylserine was indeed observed in A431 cells treated with FVCR in this study. Depolarization of mitochondrial outer membrane forms an integral event during apoptosis [31] as this leads to permeability and release of cytochrome *c*, formation of apoptosome, activation of caspase cascade, and then to apoptosis. Loss in mitochondrial membrane depolarization and also generation of ROS were noticed in A431 cells upon treatment with FVCR.

In the apoptotic cells, DNA is cleaved into a population of low molecular weight (180–200 bp) fragments. Amassing of ladder patterned extra-nuclear fragmented DNA, due to inter-nucleosomal cleavage associated with apoptosis were accounted for FVCR-treated A431 cells. The results of the present study reveal the anticancer activity of vincristine from endophytic fungus *Eutypella* spp - CrP14. Our results demonstrate that FVCR possesses apoptotic induction properties in A431 cells, through a pathway that includes mitochondrial membrane depolarization, ROS generation and DNA fragmentation, and thereby inhibits growth and proliferation of A431 cells.

## Conclusions

The present study focuses on endophytic fungus isolated from *C. roseus* as potential source of vincristine production. This is the first report which demonstrates that *Eutypella* spp - CrP14 produces vincristine that has antiproliferative and apoptotic effects on SSC – A431 cell line. *Eutypella* spp - CrP14 produces about 53 ± 5.0 μg/l of vincristine when cultured under the conditions described in this study. The endophytic fungus *Eutypella* spp - CrP14 can be considered as an ideal model system to investigate and engineer the biosynthetic pathways biotechnologically, which may lead to enhanced production of pharmaceutically important vincristine. Further work is needed to improve the FVCR yield of *Eutypella* spp - CrP14 by biotechnological approaches such as strain improvement, genetic manipulation and culture conditions.

## Abbreviations

DCFH-DA: 2′, 7′- Dichlorodihydrofluorescein diacetate
DMEM- FBS: Dulbecco’s modified eagle’s medium – fetal bovine serum
FITC- PI: Fluorescein isothiocyanate – propidium iodide
FVCR: Fungal vincristine
MTT: 3-(4, 5- dimethylthiazol-2-yl)-2, 5- diphenyltetrazolium bromide
